# Comparison of the Cytotoxic Effect of 3D-Printed Resins, Resin-based CAD/CAM blocks and Composite Resin

**DOI:** 10.4317/jced.60987

**Published:** 2023-12-01

**Authors:** Numan Aydın, Serpil Karaoğlanoğlu, Aysun Kılıç-Süloğlu, Selen Öztürk

**Affiliations:** 1 University of Health Sciences, Gulhane Faculty of Dentistry, Department of Restorative Dental Treatment, Ankara, Turkey; 2 Hacettepe University, Faculty of Science, Department of Biology, Ankara, Turkey

## Abstract

**Background:**

This study investigated the cytotoxic effects of 3D-printed permanent resins, resin-based CAD/CAM blocks and composite resin on human gingival fibroblast (HGF-1) and mouse fibroblast (L929) cell line.

**Material and Methods:**

3D-printed permanent resins (Crowntec and Permanent Crown), resin-based CAD/CAM blocks (Vita Enamic and Brilliant Crios) and composite resin (Clearfill Majesty Posterior) were used in the study. Samples were prepared from the planned materials and kept in DMEM according to ISO 10993-12:2021 standard (3 cm2/ml). The cytotoxic effect of the materials on HGF-1 and L929 cells was examined by MTT test at the end of 24 and 72 h. Two-way analysis of variance test (ANOVA) was used to analyze cell viability data.

**Results:**

3D-printed permanent resins, resin-based CAD/CAM blocks and composite resin extracts showed similar cell viability on HGF-1 and L929 cells at the end of 24 h (*p*>0.05). Resin-based CAD/CAM block (Vita Enamic) produced the highest cell viability on HGF-1 and L929 cells at the end of 72 h (*p*<0.05). Cell viability values of samples produced in 3D printers with different printing properties did not differ significantly (*p*>0.05).

**Conclusions:**

3D-printed permanent restoration resins showed similar cell viability on HGF-1 and L929 cells to resin-based CAD/CAM blocks and composite resin.

** Key words:**3D-printed resin, CAD/CAM block, Composite resin, Cytotoxicity, Human Gingival Fibroblast.

## Introduction

Composite resins have been used for the restoration of dental tissue losses for many years (1). Today, as a result of the introduction of new digitization technologies and tools, the use of computer-aided design and production systems (CAD/CAM) has become widespread. CAD/CAM systems that meet standard production processes can process ceramic and resin-containing restorative materials (2). Recently, 3D printers, which are a method of combining materials by applying a layer on a layer to create an object from 3D model data, have started to be used in the field of dentistry (3,4). The advantages of 3D printing include short production time, less material use and no milling system (5).

Additive manufacturing based on computer-aided design models uses different printing technologies to produce designed data. Stereolithography (SLA), one of the additive manufacturing methods developed for the production of dental models, uses UV laser or UV LED to polymerize modeling regions (6). Digital light projection (DLP), unlike dynamic laser using SLA technology, uses high-power LED to polymerize the entire planar area of the model structure in two dimensions (x/y axes) at the same time (7).

In dentistry, 3D printers can produce surgical guides, occlusal splints, working models, mobile section prosthesis skeletons, full prostheses, temporary crowns and bridges using different printing resins (8). The esthetic, durability and biocompatibility properties of the resin printing material have been improved and allowed to produce permanent crown, bridge, inlay-onlay restoration.

Despite the widespread use of resin-containing materials, these materials can release monomers in their structures due to physical and chemical effects in the oral environment (9). In addition, it is reported that bisphenol-A glycidylmethacrylate (Bis-GMA), triethylene glycol dimethacrylate (TEGDMA) and urethane dimethacrylate (UDMA), which are one of the basic monomers in the organic matrix of resin-based materials, cause cytotoxic effects on cells (10).

Composite resin and resin-based CAD/CAM blocks are used clinically successfully (11,12). In the studies, it was reported that 3D printer permanent resins showed similar mechanical properties to CAD/CAM blocks (13). However, studies on the toxic effects of 3D-printed permanent restoration resins are limited. The aim of this study is to examine the cytotoxic effects of 3D-printed permanent resins, resin-containing CAD/CAM blocks and composite resin on HGF-1 and L-929 mouse fibroblast cells *in vitro* according to ISO 10993-12:2021 standards with MTT test. The null hypothesis of this study is that 3D-printed permanent resins will have a cytotoxic effect similar to resin-based CAD/CAM block and composite resin.

## Material and Methods

In our study, 3D-printed permanent restoration resins (Crowntec, Saremco Dental AG, Zwitserland and Permanent Crown, Formlabs, USA), resin-based CAD/CAM blocks (Vita Enamic, Vita Germany and Brilliant Crios, Coltene, Germany) and composite resin (Clearfil Majesty Posterior, Kururay, Japan) were used. The materials used and their properties are shown in Table 1.

**Table 1:**
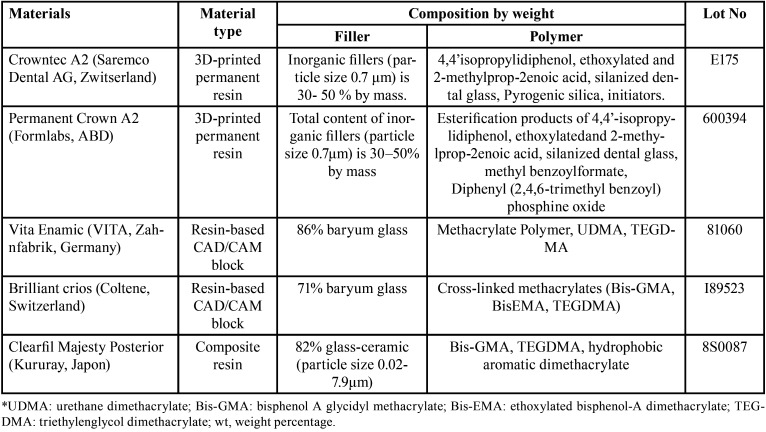
Resin-based CAD/CAM blocks, 3D-printed resins and composite resin used in the study.

-Preparation of samples

DLP 3D printer (Asiga MAX UV, Australia) was used to prepare samples from Crowntec resin. Samples were produced in accordance with the manufacturer’s recommendations with 50 µm layer printing thickness and sizes of 12×8×2 mm3. Then, the produced samples were cleaned with 99% isopropyl alcohol. Post polymerization process of the samples was carried out in polymerization device (Labolight DOU, GC, Japan) for 6 minutes.

SLA 3D printer (Form3B+, Formlabs, MA, USA) was used in the preparation of samples from Permanent Crown resin. Samples from Permanent crown resin were produced in 12×8×2 mm3 sizes with a layer thickness of 50 µm. After the printing process, the samples were cleaned for 3 minutes with an automatic washing machine (FormWash, Formlabs, MA, USA) containing isopropyl alcohol. Post-polymerization was performed with FormCure (Formlabs, MA, USA) at 60°C for 20 minutes as recommended by the manufacturer.

Precision cutting machine (MICRACUT 201, Bursa, Turkey) was used to prepare samples from resin-based CAD/CAM blocks. Samples of 12×8×2 mm3 were prepared using the precision cutting machine at low speed (150 rpm) under water cooling.

The samples from the composite resin (Clearfil Majesty Posterior, Kururay Noritake, Japan) were prepared using (12 x 8 x 2 mm3) silicone mold. Glass lamella was placed on the composite resin placed in the silicone mold using a spatula and polymerized with an LED light device. LED light device was used in the polymerization process.

Two-stage finishing and polishing systems (Clearfil Twist Dia, Kuraray, Japan) were used for polishing process of the samples prepared from 3D-printed permanent resins, resin-based CAD/CAM blocks and composite resin. Finishing and polishing on both surfaces of the samples were carried out under water cooling for 20 seconds at a speed of 10.000 rpm. After polishing, all samples were cleaned with ionized water for 10 seconds.

Preparation of extracts

The extracts from the samples of the resin-containing restorative materials planned in the study were prepared according to ISO 10993-12:2021 (14). After the prepared samples were placed in 24-meshed plates (3 cm2/ml), 5 mL of Dulbecco’s Modified Eagle Medium (DMEM) was added and incubated for 72 h at 37°C in the dark. After incubation, the original solutions were used in cell culture in a 1:1 ratio after the 22 µm filter was sterilized.

-Cell Culture

Human gingival fibroblast cell lines (HGF-1, American Type Culture Collection, Manassas, VA) were routinely transplanted at 37°C and 5% CO2 in DMEM supplemented with 10% fetal bovine serum, 1% penicillin and streptomycin. The cells were incubated for 24 h with 1 x 104 cells/well in 96-well plates. The L929 fibroblast cell line used in the study was removed from storage at -196°C and dissolved in a water bath at 37°C and centrifuged. The cells were routinely maintained in DMEM supplemented with 10% fetal bovine serum (PAA Laboratories, Linz, Austria), 1% penicillin and streptomycin at 37°C and 5% CO2 in a humidified incubator. The cells were incubated for 24 h with 1 x 104 cells/well in 96-well plates.

Spectrophotometric readings indicate the level of cellular metabolic activity. This activity represents inhibition of succinyl dehydrogenase activity through contact between cells and extracts of resin-containing materials. In the study, the extracts of the resin-containing materials (1:1) were incubated on the cells for 24 h and 72 h at 100 µL. It was then washed with phosphate-buffered saline (PBS) to neutralize other effects of extracts of resin-containing materials on cells.

-Cytotoxicity Test

Cell viability rate was determined using MTT analysis (3-(4,5-dimethylthiazol-2-yl)-5-(3-carboxymethoxyphenyl)-2-(4-sul-fophenyl)-2H-tetrazolium) (Sigma-Aldrich, St Louis, USA). 13 µL of MTT solution was added to each well and the cells were incubated for 3 h. The resulting formazan crystals were dissolved by removing the culture medium and adding 100 µL of dimethyl sulfoxide solvent (Sigma-Aldrich) to each well. The plates were shaken at room temperature for 10 minutes to dissolve the crystals, and then enzyme inhibition in a microplate reader was measured using a spectrophotometer (BIO-TEK μQuant plate reader) at 550 nm. The experiment was repeated three times. The percentage of cell viability in the experimental groups was calculated by accepting 100% of the viability in the control group.

-Statistical analysis 

In the study, SPSS 22.0 (SPSS Inc., Chicago, IL, USA) program was used to analyze the cell viability data of the materials. The normality distribution of the data was evaluated by Kolmogorov-Smirnov and Shapiro-Wilk tests. Viability data of normally distributed HGF-1 and L929 cells were evaluated using two-way analysis of variance (ANOVA) and Tukey post hoc test (*p* <0.05).

## Results

**Figure 1:**
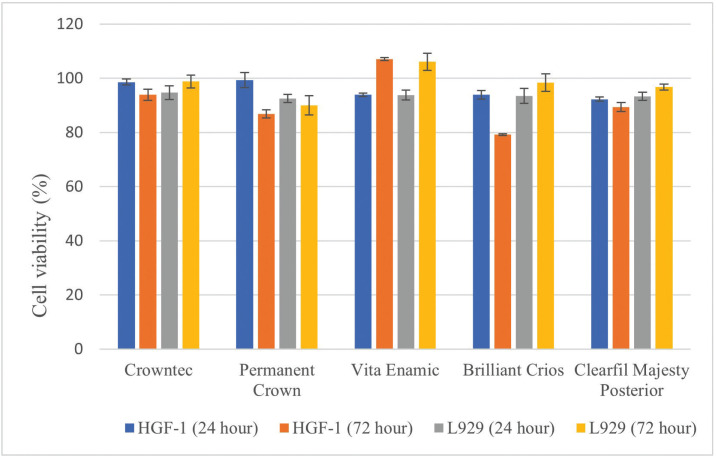
MTT test results of the cytotoxic effect of 3D-printed permanent resins, resin-based CAD/CAM blocks and composite resin on HGF-1 and L929 cells.

**Figure 2:**
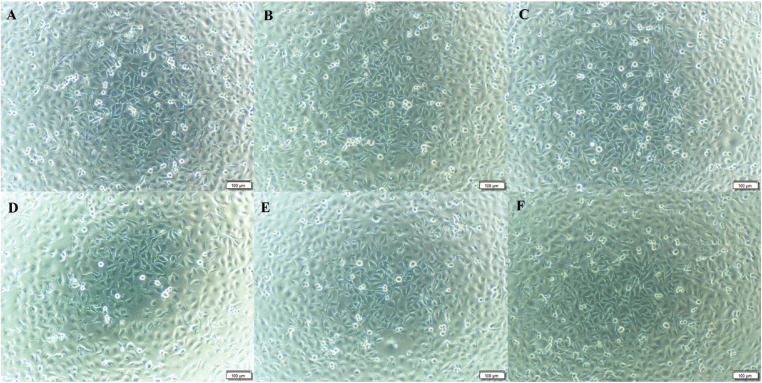
Microscope view of MTT test results after 72 hours for the cytotoxic effect of 3D-printed permanent resins, resin-based CAD/CAM blocks and composite resin on HGF-1. A: DLP 3D-printed resin (Crowntec), B: SLA 3D-printed resin (Permanent Crown), C: Resin-based CAD/CAM blok (Vita Enamic), D: Resin-based CAD/CAM blok (Brilliant Crios), E: Composite resin (Clearfil Majesty Posterior) and F: Control.

When the cell viability values of the extracts of the resin-containing restorative materials used in the study on HGF-1 cells at the end of 24 h were examined, no statistically significant difference was observed between 3D-printed permanent restoration resins, resin-based CAD/CAM blocks and composite resin (*p*>0.05), (Table 2, Fig. 1). Hybrid resin-based CAD/CAM block (Vita Enamic) showed the highest cell viability on HGF-1 cells at the end of 72 h (*p*<0.05), (Fig. 2). Although the samples prepared in the 3D-printed produced more cell viability than the composite-reinforced CAD/CAM block (Brilliant Crios) at the end of 72 h, there was no statistically significant difference with the composite resin (Clearfil Majesty Posterior) (*p*>0.05). Extracts of samples produced from permanent resin in SLA and DLP 3D printer showed similar cell viability on HGF-1 cell (*p*>0.05)

**Table 2:**
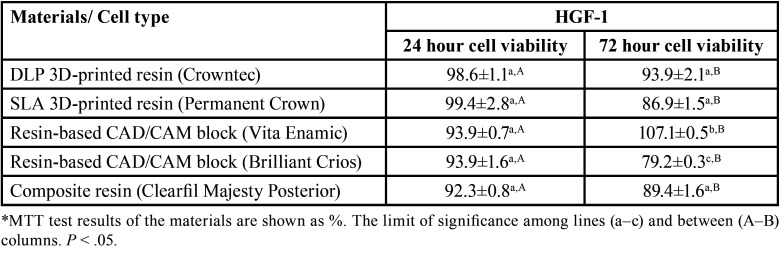
MTT test results of the cytotoxic effect of 3D-printed permanent resins, resin-based CAD/CAM blocks and composite resin on HGF-1 cells.

3D-printed permanent restoration resins, resin-based CAD/CAM blocks and composite resin extracts produced similar cell viability on L929 cells at the end of 24 h (*p*>0.05), (Table 3, Fig. 1). The extracts of the samples prepared from the hybrid resin-based CAD/CAM block (Vita Enamic) produced more cell viability on L929 cells at the end of 72 h (*p*<0.05), (Table 3, Fig. 1). Samples prepared in the DLP and SLA printer from permanent resin produced similar cell viability at the end of 72 h on L929 cells (*p*>0.05). In addition, there was no statistically significant difference between the extracts of the samples prepared in composite resin, resin-based CAD/CAM block and 3D-printed resin (*p*>0.05).

**Table 3:**
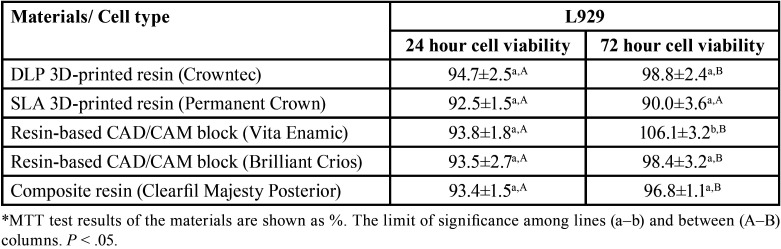
MTT test results of the cytotoxic effect of 3D-printed permanent resins, resin-based CAD/CAM blocks and composite resin on L929 cells.

3D-printed permanent restoration resins reduced cell viability over time on resin-based CAD/CAM blocks and composite resin HGF-1 cells (excluding hybrid CAD/CAM block). On L929 cells, the extracts of the samples prepared in the hybrid CAD/CAM block and DLP 3D printer did not reduce cell viability. All materials used in the study showed cell viability above 70%.

## Discussion

Resin-containing restorative materials that are in long-term contact with keratinized epithelium and soft tissues in the mouth can have a toxic effect as a result of the release of monomer from their structures after polymerization (15). In this study, the toxic effects of 3D-printer permanent restoration resins, resin-based CAD/CAM block and composite resin on HGF-1 and L929 mouse fibroblast cells were examined. Our null hypothesis was accepted because of 3D-printed permanent restoration resins showing cytotoxic effects similar to resin-based CAD/CAM blocks and composite resins.

Although various test methods are used in studies evaluating the biocompatibility of materials, animal experiments and cell culture tests, which are among the restorative materials used in dentistry, are widely preferred (16). To evaluate the cytotoxicity of dental materials, ISO 10993-5:2009 proposed several cell culture test models (17). These are direct contact (direct method), indirect contact with a barrier (indirect method) and the method in which materials extracts are added to cells (extract method). Lim *et al*. (18) compared these *in vitro* test models used to evaluate the cytotoxicity of composite resins and recommended extract test due to higher sensitivity if a single test model is to be used. In our study, all materials also used the extract test method on HGF-1 and L929 mouse fibroblast cells.

The construction of dental materials that incorporate resin typically uses monomers like BisGMA, UDMA, and TEGDMA (19,20). Although similar monomers are added to the structure of the resins prepared for production in 3D printer, BisGMA and UDMA have high molecular weight and high viscosity. Low-viscosity resins are generally preferred to ensure the accuracy of restorations produced in 3D printers (21). Therefore, non-hydroxylated monomers with lower viscosity, such as ethoxylated bisphenol-A-dimethacrylate (BisEMA), are often used in combination with TEGDMA and other diluents (22). Dimecacrylate monomers in the structure of resin-containing restorative materials have been reported to be cytotoxic (10,23). Grenade *et al*. (24) reported that despite the presence of dimethacrylate resin added to the glass-ceramic network, the hybrid CAD/CAM block achieved similar results to lithium disilicate glass ceramic in terms of HGF behavior (binding, proliferation, and propagation). In another study on hybrid CAD/CAM block, unlike conventional composite resins, it did not show any direct cytotoxic effects or effects in terms of proliferation, extracellular matrix synthesis, morphology or inflammatory response (25). These results are explained by the high degree of conversion of the hybrid CAD/CAM block. In our study, hybrid CAD/CAM block (Vita Enamic) showed the highest cell viability. But the composite reinforced CAD/CAM block (Brilliant Crios) showed less cell viability than the hybrid CAD/CAM block. It is thought that the decrease in cell viability of the composite-reinforced CAD/CAM block is due to the absence of the toxic Bis-GMA monomer (26) in its structure.

Polymerization of resin-based CAD/CAM blocks under high temperature and pressure leads to a significantly higher degree of conversion compared to conventional light-polymerized composite resins. In addition, industrial polymerization forms a homogeneous material with less pores and irregularities. UV led is used in the polymerization of 3D resins. Post-production post-polymerization process increases the degree of conversion. However, it has a relatively low filler content (<50% by weight) to maintain the required low viscosities of resins in 3D printing. However, resin blocks are generally loaded with high filling content (> 70% by weight). The high filler ratio reduces the monomer fraction required for matrix and resin blocks, thus providing less residual monomer with oscillating potential (27). Wuersching *et al*. (28) reported that the industrial polymerization method is effective in showing more positive results in terms of cytotoxicity of resin-based CAD/CAM blocks (Tetric CAD and Telio CAD) compared to 3D resins. In our study, although 3D permanent resins showed less cell viability than resin infiltrated CAD/CAM block, composite-reinforced block created more cell viability.

It has been observed that 3D printing type and printing parameters can change the mechanical properties of 3D printing and this can change its subsequent biological properties (28). Atria *et al*. (13) have stated that 3D resins exhibit similar cell behavior on HGF cells. In our study, pre-processing, cleaning and post-polymerization were carried out according to the manufacturer’s instructions. Although the samples produced in DLP 3D printers showed more cell viability on HGF and L929 cells than the samples produced in SLA 3D printer, there was no significant difference.

Non-polymerized monomers in the deeper layers of 3D printed resin can separate from the structure over time and create a cytotoxic effect (29). Kim *et al*. (30) reported that it is consistent with the findings that 3D-printed crowns and bridge resins exhibit increased cytotoxicity after 48 h of incubation compared to after 24 h. This is also consistent with the results obtained by Bayarsaikhan *et al*. (31) who stated that cytotoxicity increased rapidly with the increase in incubation time from 24 h to 48 h and 72 h. In our study, 3D resins showed more cytotoxic effects on HGF-1 cells after 72 h, compared to 24 h results.

Cell viability is measured as a percentage compared to the number of viable cells in the negative control in studies assessing cytotoxicity. A material has cytotoxic potential, in accordance with ISO 10993-5, if its cell viability is lower than 70%. It was reported that composite resins did not show any toxic effect on L929 cells (32). Resin-based materials used in the study showed over 70% cell viability.

Within the limitations of this *in vitro* study, permanent restoration resins prepared in 3D printer showed similar cytotoxic effects to resin-based CAD/CAM block and composite resin. However, in the studies conducted, the microhardness values of 3D permanent restoration resins are lower than resin-based CAD/CAM block and composite resin. In particular, the aging process can lead to the release of monomers from resin-containing materials. At the same time, exposure to different physical and chemical factors in the oral environment may change the toxic effects of these materials. Since the elution procedure in this study is performed with fresh samples and therefore our findings are only valid, it limits the importance of our results. It would be beneficial to conduct studies on the long-term cytotoxic effects of 3D-printed permanent resins.

## Conclusions

3D-printed permanent restoration resins showed similar cell viability to the resin-based CAD/CAM blocks and composite resin on HGF-1 and L929 cells. 3D printing type did not affect cell viability values on HGF-1 and L929. 3D-printed permanent restoration resins did not have a toxic effect on HGF-1 and L929 cells.
